# From systems to biology: A computational analysis of the research articles on systems biology from 1992 to 2013

**DOI:** 10.1371/journal.pone.0200929

**Published:** 2018-07-25

**Authors:** Yawen Zou, Manfred D. Laubichler

**Affiliations:** 1 Center for Biology and Society, School of Life Sciences, Arizona State University, Tempe, Arizona, United States of America; 2 School of Humanities and Social Science, Chinese University of Hong Kong, Shenzhen, Shenzhen, Guangdong Province, China; University of Alberta, CANADA

## Abstract

Systems biology is a discipline that studies biological systems from a holistic and interdisciplinary perspective. It brings together biologists, mathematicians, computer scientists, physicists, and engineers, so it has both biology-oriented components and systems-oriented components. We applied several computational tools to analyze the bibliographic information of published articles in systems biology to answer the question: Did the research topics of systems biology become more biology-oriented or more systems-oriented from 1992 to 2013? We analyzed the metadata of 9923 articles on systems biology from the Web of Science database. We identified the most highly cited 330 references using computational tools and through close reading we divided them into nine categories of research types in systems biology. Interestingly, we found that articles in one category, namely, systems biology’s applications in medical research, increased tremendously. This finding was corroborated by computational analysis of the abstracts, which also suggested that the percentages of topics on vaccines, diseases, drugs and cancers increased over time. In addition, we analyzed the institutional backgrounds of the corresponding authors of those 9923 articles and identified the most highly cited 330 authors over time. We found that before the mid-1990s, systems-oriented scientists had made the most referenced contributions. However, in recent years, researchers from biology-oriented institutions not only represented a huge percentage of the total number of researchers, but also had made the most referenced contributions. Notably, interdisciplinary institutions only produced a small percentage of researchers, but had made disproportionate contributions to this field.

## Introduction

Systems biology has emerged over the past two decades, aiming to examine organisms from a systematic and holistic perspective. It is characterized by large datasets and modeling. The development of high-throughput technologies in the 1990s brought forth a deluge of data to biology. Without mathematical models and computational simulations, however, the data could not be understood at the time. In the early 1990s, systems biology had strong mathematical and computational traditions, and was drastically different from traditional biological disciplines such as evolutionary biology, molecular biology, and development biology. Early systems biologists have made many attempts to improve on the methodology such as developing various algorithms for dealing with large datasets and formalisms that model a specific network including gene regulatory networks, signal transduction networks, and metabolic networks [1]. With these developments many sub-disciplines of systems biology have been established, such as systems pharmacology [2], which explores the mechanisms behind drugs by means of systems biology methods, and cancer systems biology, which is a field that applies computational modeling to reveal the carcinogenic pathways [3]. A systems biology approach offers many advantages to biologists, especially medical scientists because first, behind many complex diseases such as cardiovascular disease, respiratory diseases, and diabetes, there are complex biological networks, instead of one or several genes. Second, the diagnose and cure for complex diseases requires a systematic understanding of these complex networks, and systems biology approach makes the modeling, perturbation, and prediction of complex networks possible. Because the above reasons, systems biology approach offers a proactive instead of reactive approach [4].

Systems biology is an interdisciplinary field, where biologists, referred to here as biology-oriented scientists, including evolutionary biologists, developmental biologists, molecular biologist, pharmacists, etc., and engineers, computer scientists, and mathematicians, referred to as systems-oriented scientists, work together. Systems-oriented scientists have contributed greatly to the advancement of systems biology. For example, Hiroaki Kitano published the most highly cited article in systems biology, edited the first monograph on systems biology, founded the Systems Biology Institute in Tokyo, and organized the first International Conference of Systems Biology in Tokyo in 2000 [5,6]. Kitano was trained as an engineer and is the head of Sony Computer Science Laboratories. Another example is Albert-Laszlo Barabási, whose work on network theory has won him many awards in systems biology. He was trained as a physicist, yet he publishes widely in systems biology (e.g. [7]).

As increasing number of tools provided by systems biology prove to be more and more powerful, more and more biologists are joining the field of systems biology. In the past, the modelers were often from mathematics or engineering backgrounds, and had little training in biology. Nowadays the trend is that modelers have realized that biological systems are not simply analogical to physical systems or chemical systems, and therefore they need to understand the functions and mechanisms of biological systems [8]. This has led to more and more modelers and biologists working together, with the models playing a role in simulation, prediction, and guidance of real experiments [9].

Scientists from different backgrounds have different epistemologies and methodologies, thus the subjects of their research inevitably differ, with some being biology-oriented and others systems-oriented. One may therefore wonder how biology-oriented and systems-oriented research topics changed within systems biology. This study examines the history of systems biology from 1992 to 2013. Some scholars argue that systems biology’s early roots emerged in the middle half of the twentieth century such as theories about cybernetics proposed by Norbert Wiener in 1948, Denis Noble’s heart model in 1960, and Ludwig von Bertalanffy’s General Systems Theory [10,11,12]. We argue that these early work failed to establish systems biology as a legitimate discipline so we did not choose to an older time. We chose the year 1992 instead of a more recent time because although there were very few articles containing the term “systems biology” in the 1990s, we argue that technologies that enabled the generation of big data and algorithms for the modeling of complex systems had been developed in the 1990s. Conceptually, modern systems biology had its early roots in the early 1990s. Leroy Hood wrote in a 1992 book that “the future of biology will depend upon the analysis of complex systems and networks” [13].

We utilized several computational tools to analyze the metadata of the systems biology literature to answer the following questions: From 1992 to 2013, how did the topics of systems biology research change and did this change reflect a shift towards more biology-oriented topics? From what types of institutions did the systems biologists come and how did their institutions change over time? Our research allows system biologists and biologists in other fields to better track the history, dynamics, and future trends of this discipline. For policy makers, our research is also interesting to them because this study showcases the knowledge about and tools to investigate the institutional backgrounds of the practitioners in systems biology.

## Materials and methods

### Data

The growing number of publications in a scientific field like systems biology makes it hard to identify trends and study frontiers simply by analyzing a few key papers. Bibliometrics can provide analysis tools to address these difficulties. Scholars have applied bibliographic analysis to study the history of business, science, art, and engineering [14]. Bibliographic analysis is a good way to assess the influence and quality of literature by deciding which work gets cited most and which author has the most citations [15]. Earlier attempts to analyze citation data from the Web of Science (WoS), such as those by Science, Technology, and Society scholar Susan Cozzens in the 1990s, were limited as many computational tools for the analysis of big data were not yet available [16]. However, after more than a decade of development, information scientists have produced many tools and approaches for citation analysis, which can overcome the difficulty faced by Cozzens. These include the ISI citation index, CiteSpace, HistCite, VOSViewer, to name a few [17,18,19].

In the WoS database, we searched for documents containing the term “systems biology” in their topics (including titles, abstracts, and keywords) and published from 1992 to 2013. Then by selecting those published in English, we narrowed the sample down to 9923 articles, which included research articles, reviews, editorial materials, proceeding papers, and meeting abstracts. One thing to note is that the first article in our query that contains the term “systems biology” was published in 1997 and there were few articles in the 1990s. Despite this limitation, we can use bibliographic analysis tools to get the relevant references of those 9923 articles. If one article cites about 20 references on average, then the total number of references are about 200,000. One such tool is CiteSpace, an open-access bibliometric tool, which can help analyze the references and also count which references were cited most by those 9923 articles automatically using their built-in functions [20]. The 9923 articles can be called the “research front” and the references of those 9923 articles can be called the “intellectual base” of systems biology.

We downloaded the bibliographic information for these 9923 articles. All data files are available from the Figshare database (accession number 10.6084/m9.figshare.5422594). For every article, WoS can bulk export all the bibliographic information, such as authors, title, abstract, references, and publishing year. To answer our questions, we first picked out the most cited references, and then analyzed the research categories of the most cited articles using close reading. Next, we used machine learning techniques to study the topics of systems biology embedded in the thousands of abstracts.

#### Analysis of the most cited references for the types of research (examining the intellectual base of systems biology)

The built-in functions of CiteSpace can analyze the citation frequencies of each reference, and produced a list of the most highly cited references from the year 1992 to 2013 automatically. We divided the years from 1992 to 2013 into 11 time slices, and picked 30 articles for each time slice, which included both research articles and reviews. We chose the number 30 because if we had chosen less than 30 references for each time slice, we could not have obtained statistically significant result, while choosing a sample size larger than 30 was not feasible because in early years there were not so many cited articles. For a full list of these articles, see S1 Table. After CiteSpace picked out 330 references, we manually searched for and downloaded the full texts for each reference, and then carefully read them to determine whether the articles were systems-oriented or biology-oriented and how the number of articles in each category changed over time.

This requires certain criteria. Because systems biology is an interdisciplinary science, there is no 100 percent biology-oriented or systems-oriented research. However, because any research has to be focused on a certain area, it is possible to classify these articles into a few categories, and for each category, it is easier to say whether each is more systems-oriented or biology-oriented. For previous bibliographic analysis, researchers have categorized publications of a field in categories to show the “big picture” of that field [14,21]. We have to admit that it is a formidable task to categorize publications of a scientific field as diverse as systems biology. For our analysis we derived our categories from within the literature (based on close reading) and also from categories identified by other historians and philosophers of biology.

The historians and philosophers of science Ulrich Krohs and Werner Callebaut once argued that three roots of systems biology must be discerned to account properly for the structure of systems biology, namely, pathway modeling, biological cybernetics, and -omics [22]. We agree that pathway modeling and -omics have fueled the advancements of systems biology during the past two decades. Cybernetics may be very important in the mid-twentieth century and contributed to systems biology’s theory as a root. However, in the literature on systems biology published over the last two decades, it is hard to see the influence of cybernetics as comparable to that of pathway modeling and -omics. We argue that Krohs and Callebaut’s categorization is simplistic. Systems biology is a very interdisciplinary field and has many diverse areas; therefore, we used more categories than just the three proposed by Krohs and Callebaut, and most of the articles in systems biology fall in a rather straightforward way into the nine categories we propose in Table 1.

**Table 1:** Nine categories and their descriptions.

Categories	Description	Systems-oriented or Biology-oriented
Metabolic Flux Analysis	Measures the stoichiometric data of metabolites, and relies on modeling using non-differential equations and a few parameters.	Systems-oriented
Development of high-throughput technologies	These technologies include sequencing technologies, protein chips, DNA arrays, and mass spectrometry, etc.	Biology-oriented
Algorithms, equations, and modeling	This category includes development of algorithms, equations, modeling, and simulation techniques that relies heavily on mathematical knowledge.	Systems-oriented
Omics research characterizing a real biological system	Omics research relies on data produced by high-throughput technologies and modeling; the ultimate goal is offering a system-level characterization of a real biological system.	Biology-oriented
Database development	This category involves the launch of databases storing genes, pathways, proteins, etc. It also involves standardization of data and procedures, such as the Systems Biology Markup Language.	Systems-oriented
Software development	Software is developed to process, analyze, and visualize large data.	Systems-oriented
Network properties	These properties include robustness, dynamics, stochasticity, and emergent network properties that can be applied to every system, not just biological systems. The work is mostly mathematical and theoretical.	Systems-oriented
The applications of systems biology	Systems biology is especially useful in tackling complex diseases like cancer, and has applications in bioengineering and synthetic biology.	Biology-oriented
Biological Mechanisms	This category involves using systems approach to understand a specific biological mechanism.	Biology-oriented

Based on close reading of these 330 articles, we used nine categories of research and we introduce what each category means and why each is either systems-oriented or biology-oriented (see Table 1). Biology-oriented research includes the following four categories: -omics-related research, high-throughput technologies, applications in engineering and medicine, and biological mechanisms. Systems-oriented research includes network properties, software development, Metabolic Flux Analysis, database development, and algorithms, equations, and modeling. For a more detailed description of each category, please see S1 Text.

#### Analysis of topics found in abstracts using topic modeling

Through the previous step, we analyzed the categories of systems biology research through manual reading of 330 articles and how the number of articles in each category changed. However, we wanted to analyze the research categories in a larger sample size, say thousands of articles, and see if the results of the latter corroborated with our close reading. Because we could not possibly have read all of them, we relied on topic modeling. A topic here can be broadly interpreted as a subfield, a category, or a research type. A good thing about topic modeling is that it can analyze millions of words quickly without humans reading them [23]. Topic modeling has many applications in the humanities, social sciences, and bioinformatics, including the study of Twitter messages to identify trends and studying the corpus of thousands of research papers or newspaper articles to show how ideas in a specific field have changed over time [23,24,25]. We used topic modeling to analyze the 8809 abstracts of articles published from 2003 to 2013 that were retrieved from the WoS Citation data. The starting year was set at 2003 because before 2003, each year only had a few publications and the number of articles is positively related to the accuracy of the results of topic modeling. Out of the 9634 articles that were published from 2003 to 2013, 8809 articles have abstracts.

Topic modeling uses probabilistic models to generate topics (each topic is represented by a cluster of words) through automatic reading of unstructured natural language [26]. One of the most widely used topic model is the Latent Dirichlet Allocation (LDA) model.

The main mechanism of topic modeling is as follows: First, the model sets a fixed number of topics and a fixed number of words in each topic. The basic idea is to view a document as a distribution of topics and a topic as a distribution of words. Second, the model randomly assigns the words in a document to a topic and calculates two probabilities: P (topic/document) and P (word/topic). Third, the LDA model utilizes Bayesian inference to adjust the word assignments iteratively until it reaches a relatively stable state. Each iteration involves assigning a word to a topic, and updating the P (word/document) and P (topic/document) to infer and update P (word/topic) [26]. The actual modeling process utilizes more complicated algorithms.

There are many tools that can implement topic modeling, for example the MALLET (Machine Learning for Language E Toolkit) and the Stanford Topic Modeling Toolbox [27,28]. We used MALLET, a software developed by Andrew McCallum at the University of Massachusetts, and it is based on an LDA model and a set of different algorithms. Words like “doi,” “paper,” “ab,” “research,” “results” and “elsevier” were compiled in an extra list of stopwords, which are words that MALLET ignores, in addition to the default one that MALLET includes. The number of topics was set at 20.

We can also analyze how the topics change over time. To offer a historical perspective, identifying topics in thousands of articles is an interesting task, but it is even more useful to see the topic trends over time [23]. MALLET also returned the composition of topics in all the documents over time. In our case, each document is an abstract of an article. For example, for a document, MALLET returned the probabilities of each of the 20 topics. If every topic is equally represented in articles, then the probability of each topic should be 5%. If the probability of a topic is higher, that means that this document has a higher probability of containing that topic. For example, if an article has a topic whose probability is higher than 40%, then this suggests that this article is indeed related to that topic. Similarly, if an article has a topic whose probability is only 1%, then it is unlikely that this article contains that topic. In our study, we consider a probability of 10% to be the threshold for considering that the topic is significant. A Python code was written to calculate the number of articles that contained a topic the probability of which was higher than 10% for a certain year. We then calculated the percentage of that number for all the articles published in that specific year.

### Analyzing the institutions of authors

We used Tethne to determine which authors were the most highly cited over the years (30 authors for each of the 11 time slices). Tethne is a Python package developed by Erick Peirson at the Digital Innovation Group at Arizona State University for bibliographic and corpus analysis. The tool was written in Python, and it is open source. It works with WoS, JSTOR, and Scopus data to visualize patterns and trends in the scientific literature.

Using Tethne, we analyzed the references for all 9923 articles. Each reference includes the information of the first author’s name and the publishing year. For each time slice, we calculated the number of the references an author published in that time slice, and arranged the authors based on that number. Then we picked out the top 30 authors with the highest number of references in each time slice for the analysis in the next step.

We were not only interested in the most highly cited 330 authors, but all the authors of those 9923 articles. Therefore, we retrieved each author’s affiliation at the time of publication from either WoS data or Google Scholar. We analyzed these affiliations to determine which category of institution the author was affiliated with. The reason we chose their affiliations instead of other information was a trade-off between the content and the accessibility of that information. For example, a person’s description on one’s own website may be a more accurate assessment of what one is doing, but this information is hard to get for thousands of authors, and harder to compare in an objective way. Affiliations are easier to obtain and can accurately reveal the institutional backgrounds for researchers of systems biology.

We built a word list by retrieving an identifying word from the affiliations of the most highly cited 330 authors. For example, if a department name has the word “anatomy,” it is likely to be a biology-oriented institution, and we used “anatomy” as an identifying word. Next, we developed a model to study the affiliations of the 9634 first authors of articles published from 2003 to 2013 in the WoS database. The reason for starting from 2003 was that in that year, 118 articles were published whereas in the previous year, only 30 articles were published. In statistics, 30 is usually considered the minimal sample size. Because we started with the year 2003, the articles we looked at was 9634 instead of 9932. Based on the word list generated by analyzing the 330 authors, we wrote a Python code to design a model that automatically labels the institutions of thousands authors.

The model is described as follows: the automatic labeling of the institutions was based on the first word, usually the department name that matched the word list. For example, in the affiliation of “Max Planck Inst Mol Plant Physiol, D-14476 Potsdam, Brandenburg, Germany,” the first word that matched the word list was “plant,” so it was labeled automatically as a “biology-oriented” institution. For institutions names that contained a word that indicated the institution’s orientation but is not on our list, we labeled them manually and added the identifying word to our word list. We then ran the process iteratively. However, some institutions were still hard to define because the affiliations retrieved from the WoS citation data did not have a specific department and only the university, so we labeled these manually as “unidentifiable,” for example, affiliation like “Los Alamos Natl Lab, Los Alamos, NM 87545 USA” would be labeled as unidentifiable. Another situation where an institution was hard to identify was that it is a foreign institution with a name like “Tech Univ Dresden, Inst Lebensmittel & Bioverfahrenstech, D-01069 Dresden, Germany.” The word list that we used to identify institution categories contain 193 words (See S2 Table).

## Results

### Research categories of the most highly cited references in the intellectual base

**Fig 1:**
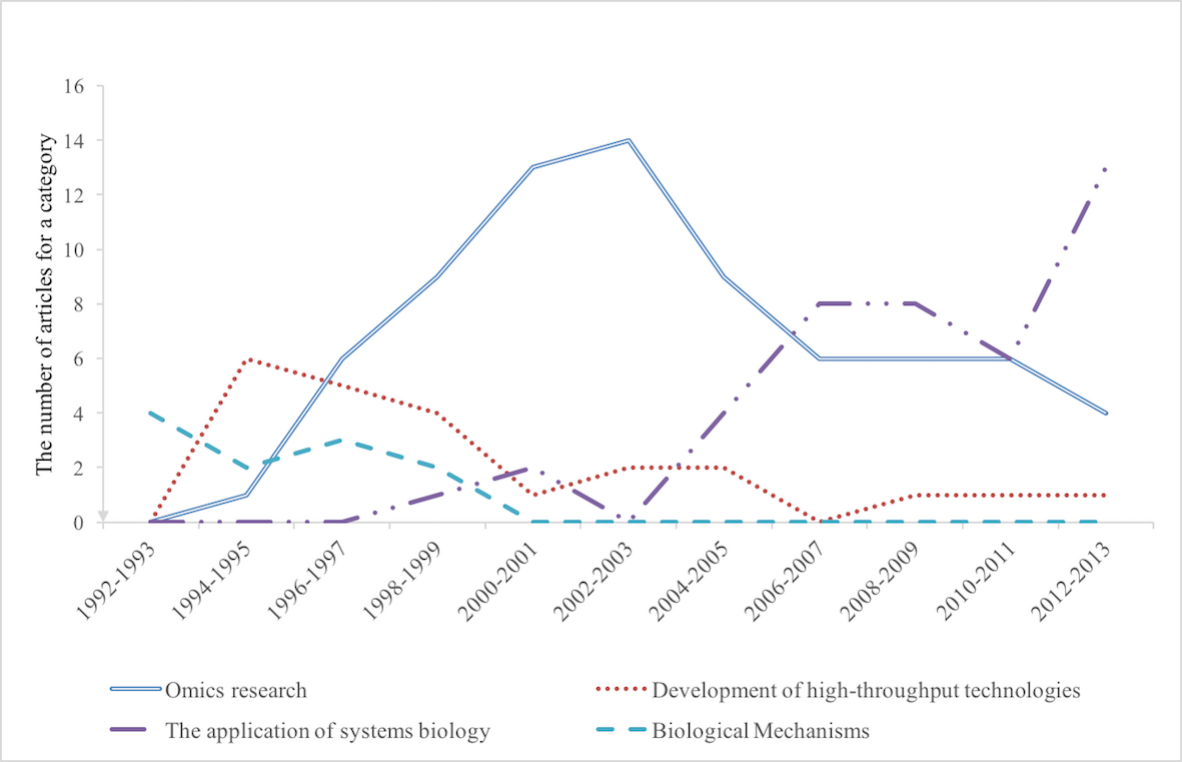
The number of the most highly cited articles in biology-oriented research categories. The *y* axis stands for the number of articles among 30 most highly cited articles for a category.

**Fig 2:**
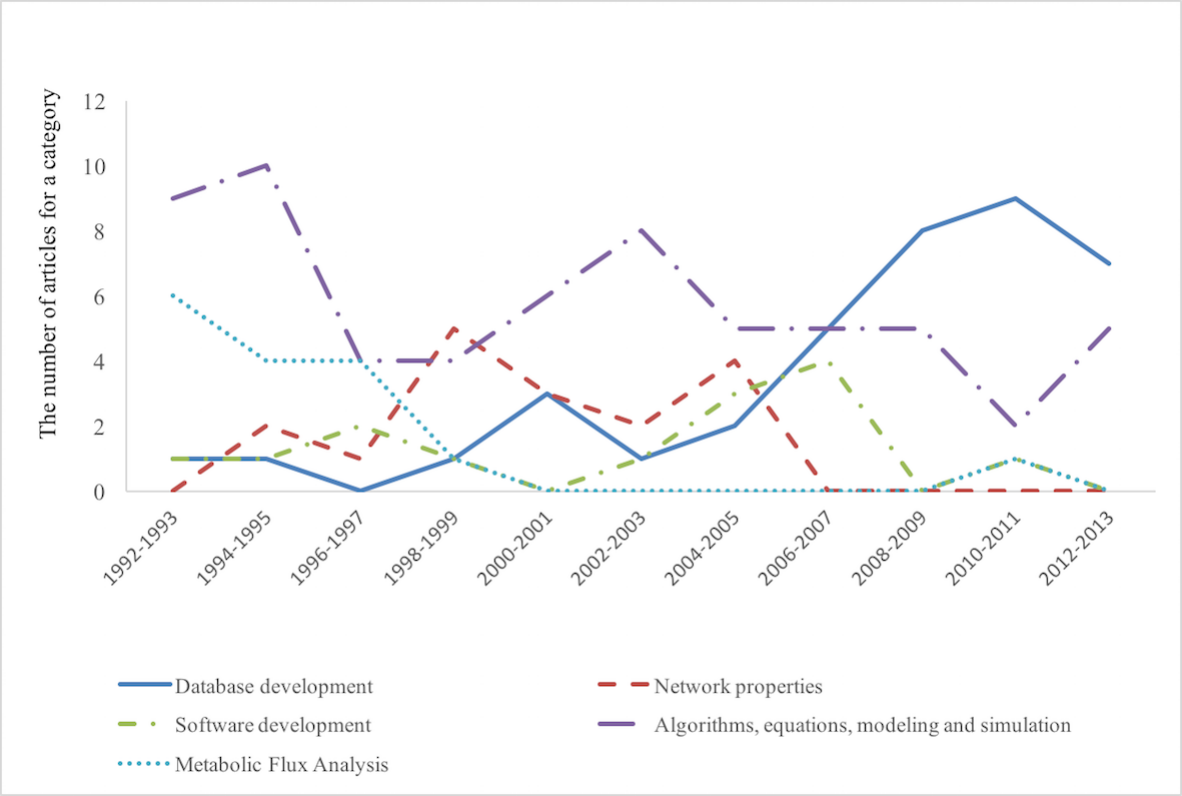
The number of most highly cited articles in systems-oriented research categories. The *y* axis stands for the number of articles among 30 most highly cited articles for each time slice.

We selected the most highly cited 30 references in 11 time slices from 1992 to 2013 (a total of 330 references). Excluding books and references that are hard to label, the results of these 330 references that fall either into biology-oriented or systems-oriented categories are depicted in Figs 1 and 2, respectively. Out of the 330 articles, CiteSpace picked out 14 references that could not be placed into these nine categories. They either focus only on topics tangentially related to systems biology, or are hard to put into any category, so they were not included in the nine categories. Each line represents a category. Fig 1 shows the number of references in four biology-oriented categories. The development of high-throughput technologies (the red line) is the category that has the highest number of articles in early stages, but this category decreased afterwards. This suggests that many early biologists involved in systems biology by developing new technologies.

Omics research (the solid blue line) began to emerge in the late 1990s and peaked around 2002. Omics research has changed from simply getting the sequence of a genome in the 1990s to actually mapping the interactions of biological molecule, be it proteins, or genes, or metabolites after 2000. An example of an article in this category is the comprehensive study of protein-protein interactions in yeast. Peter Uets and his colleagues discovered 957 possible interactions of more than 1000 proteins [29]. After that omics research decreased while the applications of systems biology (the purple line) began to increase in early 2000s, and reached a plateau from 2006 to 2010.

Then in the last four years, the rise of systems biology’s applications in medical fields and engineering became very significant. 13 out of the 30 most highly cited articles from 2012 to 2013 belong to this category. For example, one such highly cited reference in 2010 is on systems vaccinology, describing how systems approach has changed the way scientists develop vaccines [30]. Despite the fact that vaccines work well in preventing diseases, the mechanism for how they work remained largely unknown before application of a systems approach. Scientists are now starting to use systems approaches to identify the gene regulatory network after the injection of vaccines, and predict the later responses. It can help identify high-risk individuals and prevent potential harmful consequences of vaccine failures to those individuals [30].

Fig 2 shows the number of references in five systems-oriented categories. From 1992 to 1995, two categories, namely, metabolic flux analysis (the light blue dotted line), algorithms equations and models (the purple dotted line), are the top two categories. However, only the category of algorithms, equations and models maintains the same importance, and metabolic flux analysis decreases over time. This suggests that algorithms, equations and models are still very central to systems biology research, which is claimed by other scholars [31].

Database management (the dark blue solid line) emerged around 2000 and continue to be a strong presence in later years. These include the Gene Ontology, KEGG database, BioGRID, Reactome, BiGG, IntAct, STRING, to name a few [32,33]. A database is not a place where biologists dump their data, because scientists need to figure out how to store the data, how to search for data quickly, how to manage database structure, and how to develop a standard of data format that is compatible to more databases [34]. This knowledge can be classified as data science in general, which requires the input of systems-oriented scientists.

### Topics found in the abstracts of the research front

We were not only interested in the change of research types of the most highly cited references, but also in the trend of those 9923 systems biology articles. The result of the topic modeling based on thousands of articles is similar to our manual reading of the most highly cited articles. S3 Table shows the machine learning results of topics using MALLET and the labels that we assigned by reading the words in topics. The machine learning model returns the following 20 topics: biology, models, metabolomics, diseases, proteomics, synthetic biology, database and software, cell biology, systems theory, algorithms, immune system, network properties, network, genomics, technologies and tools, drug and cancer, regulation, pathway.

**Fig 3:**
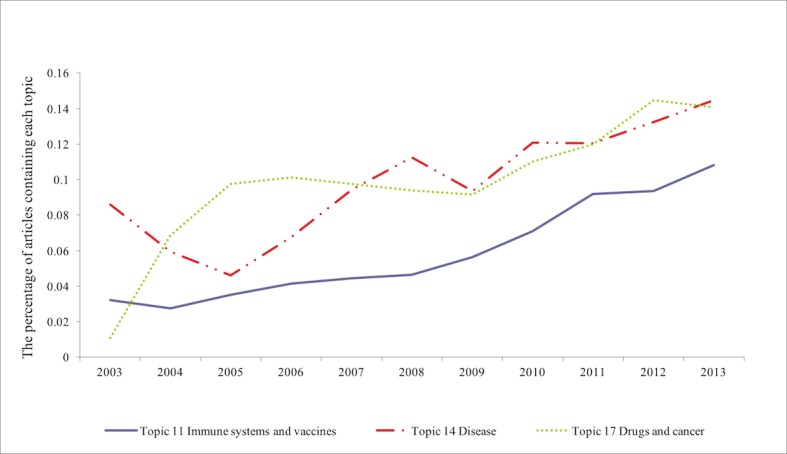
The trends of Topics 11, 14, 17. The *y* axis represents the percentage of articles published in that year that contains a topic with a probability higher than ten percent.

**Fig 4:**
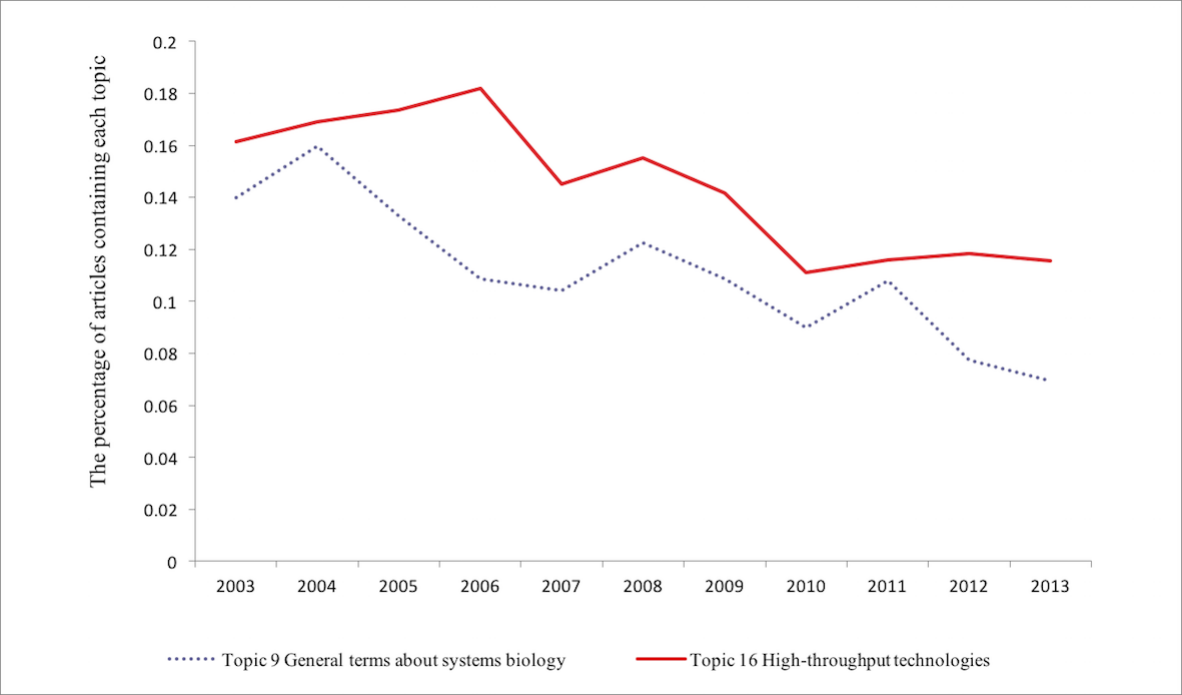
The trends of Topics 9 and 16. The *y* axis represents the percentage of articles published in that year that contain a topic with a probability higher than ten percent.

We were not only interested in what these topics are, but also the temporal change of topics. Figs 3 and 4 show the topics that have significant patterns of increasing or decreasing. Fig 3 shows that the percentage of articles that contain Topics 11, 14 and 17 increased over time. Topic 11 is about the research related to immune systems and vaccines, Topic 14 is about disease, and Topic 17 is about drugs and cancer. These are related to the applications of systems biology, which is similar to our finding about the articles in this category among the most highly cited articles based on close reading, which showed an increase.

Fig 4 shows how the percentage of articles that contained topics 9, and 16 decreases. Topic 9 includes some general terms about systems biology, and Topic 16 is about high-throughput technologies, which includes words like “high,” “throughput,” “technologies,” and “techniques.” The result is similar to our findings about the percentage of articles in the category of high-throughput technologies among the most highly cited articles, which is decreasing over the years (The percentage for articles that contained each of the 20 topics can be seen in S4 Table).

### The institutional contexts for systems biologists

Table 2 lists the affiliations of the first most highly cited authors in each time slice. The affiliations show the department, the university or institution, and geographical information such as city and country. The table shows that the most highly cited authors in each time slice changed quickly throughout the years, suggesting that systems biology was evolving quickly. The institutions they are affiliated with are very diverse and interdisciplinary. We classified the institutions of the top 30 authors from 11 time slices into four categories: biology-oriented, systems-oriented, interdisciplinary and systems biology institutions.

**Table 2:** The most highly cited authors and their institutions.

Time Slice	The most cited author in each time slice	Authors’ Institutions	Category
1992–1993	KAUFFMAN S	Department of Biochemistry and Biophysics, School of Medicine, University of Pennsylvania and Sante Fe Institute, Sante Fe, New Mexico, U.S.A.	Interdisciplinary/Systems Biology
1994–1995	BENJAMINI Y	Department of Statistics, School of Mechanical Studies, Tel Aviv University, Tel Aviv, Israel	Systems-oriented
1996–1997	HEINRICH R	Theoretical Biophysics Group, Institute for Biology, Humboldt University Berlin, 10115 Berlin, Germany	Interdisciplinary
1998–1999	HARTWELL LH	Fred Hutchinson Cancer Center, Seattle, Washington 98109, USA.	Biology-oriented
2000–2001	IDEKER T	Inst Syst Biol, Seattle, WA 98105 USA.	Systems biology
2002–2003	KITANO H	Sony Comp Sci Labs Inc, Tokyo 1410022, Japan.	Systems-oriented
2004–2005	BARABASI AL	Department of Physics, University of Notre Dame, Notre Dame, Indiana 46556, USA	Systems-oriented
2006–2007	ALON U	Department of Molecular Cell Biology, Weizmann Institute of Science, Rehovot 76100, Israel.	Biology-oriented
2008–2009	FEIST AM	Department of Bioengineering, University of California, San Diego, La Jolla, California 92093, USA.	Interdisciplinary
2010–2011	ROUKOS DH	Univ Ioannina, Biosyst & Synthet Genom Network Med Ctr BioSynGen, Ioannina, Greece.	Systems biology
2012–2013	ZHANG AH	Heilongjiang Univ Chinese Med, Natl TCM Key Lab Serum Pharmacochem, Key Lab Chinmed, Dept Pharmaceut Anal, Harbin 150040, China	Biology-oriented

Biology-oriented institutions have words like “cancer,” or “genetics” that are related to the life sciences. We noticed that a fair number of authors are from a medical institution, and even pharmaceutical companies, like Glaxosmithkline and Syngenta. Systems-oriented institutions include those related to “statistics,” “mathematics,” “physics,” “chemistry,” and other non-biology disciplines. We also found that some researchers are from companies such as Microsoft, Siemens, and Sony. “Interdisciplinary institutions” refer to those that related to interdisciplinary field such as “biochemistry,” “biophysics,” “bioengineering,” “biotechnology,” and “bioinformatics.” Systems biology institutes include those that specifically use the words like “systems biology,” or “biosystem,” or “biosyst.” For some institutions that are hard to tell which category they belong to, we did not label them and include them in the calculation.

**Fig 5:**
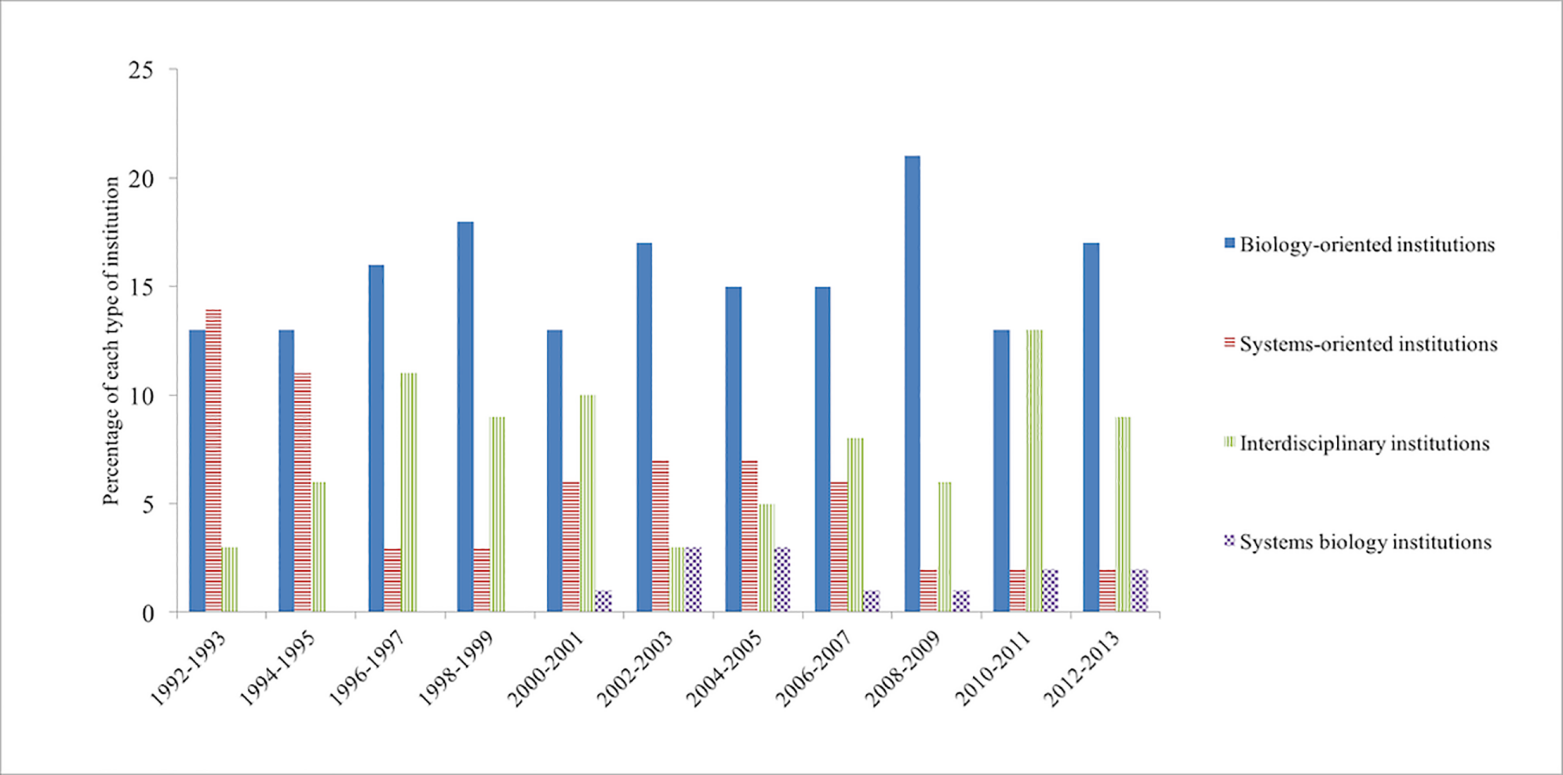
The most highly cited authors’ institutions. The *y* axis stands for the number of authors in a category among the top most highly cited 30 authors.

We used Tethne to determine which authors were the most highly cited over the years (30 authors for each of the 11 time slices). The results of the categorization of the affiliations of the 330 highly cited authors are shown in Fig 5. In each time slice, the top 30 authors’ institutions were plotted in four color-coded bars to represent the four categories. The figure shows that the number of authors from systems-oriented institutions (red bar) was first almost the same as biology-oriented institutions, but their number diminished over the years. For example, in the time frame of 1992 and 1993, one of the most cited authors is Daniel T. Gillespie, a physicist working at the Research Department of Naval Weapons Center at the time. His work on stochastic simulation algorithm in chemical kinetics contributed to the simulation method adopted by later systems biologists [35]. He contributed to the foundation of systems biology while this discipline was still in its “early roots” stage, and he did not mention systems biology in his work at the time.

Scientists from interdisciplinary institutions (green bar) have fueled the advancement of systems biology not only in the early days of systems biology, but also in more recent years. These institutions have provided a place for early systems biologists to stay. While in the 1990s, there were no systems biology institutions (purple bar), which only emerged within the slice from 2000 to 2001. In each time slice after 2001, there were a few authors coming from systems biology institutions. The number of the most cited authors from a biology-oriented institution (blue) tends to fluctuate over the years, but this category has the highest number compared to other categories in every slice except in the time slice from 1992 to 2013.

**Fig 6:**
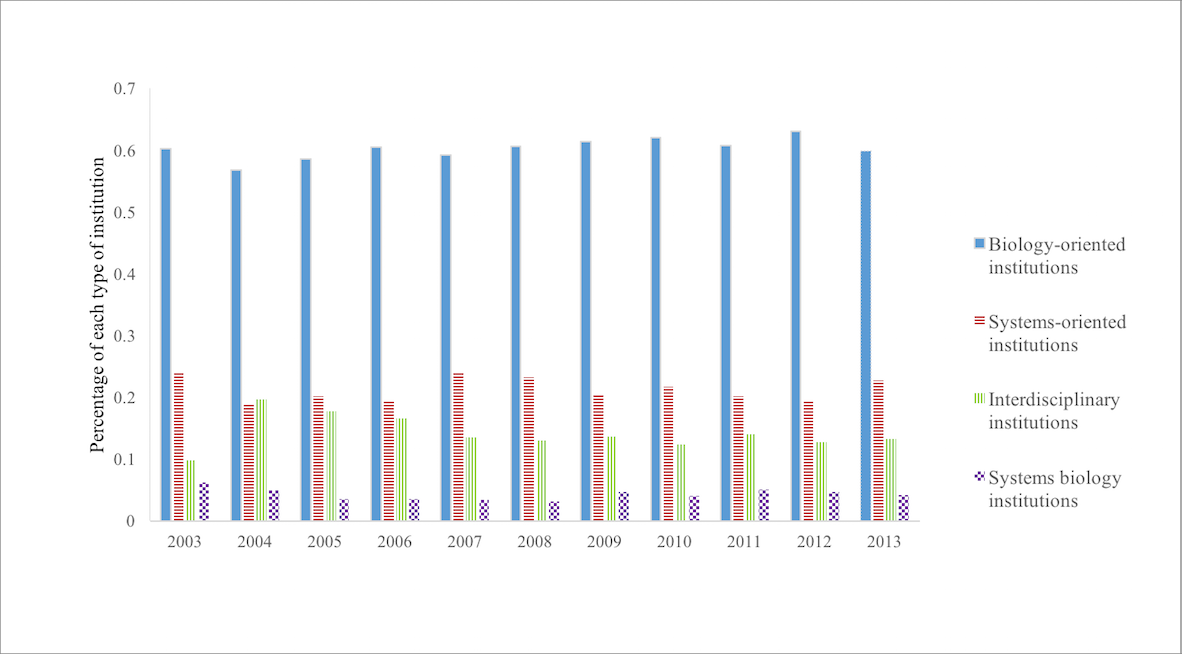
All authors’ institutions from 2003 to 2013. The number in each category changed little from 2003 to 2013. The *y* axis stands for the percentage of each type of institution.

Fig 6 shows the result of all the first authors who published between 2003 and 2013 retrieved from WoS citation data. Out of all 9876 authors who published between the years 2003 and 2013, a total of 779 (7.89%) were unidentifiable. We excluded those articles when calculating the percentage of four categories. After taking out the unidentifiable authors’ affiliations, it shows that the percentage of each category has remained quite constant, which means that from 2003 to 2013, the institutional context for systems biologists did not change much.

Between the years 2003 and 2013, on average 60.85% of authors came from a biology-oriented institution; 21.16% came from a systems-oriented institution; 13.75% were from an interdisciplinary institution; and 4.23% were from a systems biology institution. These statistics suggest that the institutional context for all authors publishing on systems biology is different than that for the people who published the most highly cited articles on systems biology. Notably, the latter had a larger percentage of authors who were affiliated with interdisciplinary and systems-oriented institutions. This is an interesting observation that the people who were cited most and the people who published in a field have different patterns in terms of their affiliations, and we will discuss that in the next section.

## Discussion

This study suggests that systems biology is becoming more and more biology-oriented, especially with its biological applications. Among the most recent highly cited articles, the articles in the category of applications of systems biology have increased tremendously. Over the years among the most-highly cited authors, fewer authors were from systems-oriented institutions. The trends of topics based on topic modeling suggested that more recently a higher percentage of articles includes topics about immune system and vaccines, diseases, drugs and cancer.

Our research findings are echoed in some other scholars’ observations about systems biology. Alan Aderem argues that “biology dictates what new technology and computational tools should be developed, and once developed, these tools open new frontiers in biology for exploration. Thus, biology drives technology and computation, and in turn, technology and computation revolutionize biology” [36]. Some other scholars also suggest that biology needs to be more important within systems biology in regard to two aspects, first, that more biologists should be involved in systems biology, and second, that more empirical biological problems need to be addressed [37]. Jane Calvert and Joan H. Fujimura carried out interviews with many researchers in systems biology, including biologists, mathematicians, physicists, and computer scientists [37]. One of the biologists said, “I think biologists need to drive systems biology, because if it’s driven by computation or engineers, without a depth of training in biology, they lose that sense, they tend to treat molecules as nodes and edges without a sense of how they’re performing their functions” [37]. Physicists and computer scientists might also agree with that, because often those who want to model a biological system sometimes have difficulty find a biological expert to help them to link the model to specific biological problems [38]. Along with the findings of the interviews, a systems biologist, Sui Huang, claims that biologists have become active players in systems biology because what they need to understand now is not a single gene or a protein, but networks of genes or proteins, and systems biology approach can help address these needs [39]. Huang’s article claims that systems biologists should divert their research “back to biology in systems biology” [39]. Today, systems biology has captured more biological phenomena, properties, objects such as various regulatory pathways, data, theories, and methods than its precursor did in the middle of the twentieth century. Biologists have seen the utility of applying a systems biology approach to understand the evolution and function of biological networks.

Our study shows a clear upward trend towards applications in systems biology. These applications have been centered on understanding cancer better, transforming drug discovery, and making preventative vaccines. System biology, compared to evolutionary biology, developmental biology, etc., can reveal the mechanisms behind complex diseases. System biology and medicine have become so closely related that this has led to the development of a new field called "systems medicine." We agree with Leroy Hood's point of view that the future of medicine will become "predictive, personalized, preventative and participatory" [40]. This also means a new era for biologists, and this study’s results indicate that the growth of this field is very fast, and we believe that in the near future we may be able to see some breakthroughs.

Our results on the institutional context of scholars who have published in this field also has implication for policy makers and funding agencies. The results indicate that among the most highly cited authors, while those from systems-oriented institutions have decreased over the years, those from interdisciplinary fields have come to contribute more to systems biology. Another interesting finding is the institutional contexts of the most cited authors and general authors, which suggest that the scholars who lead a field are sometimes different from those who publish in that same field. Therefore, it has been suggested by previous scholars that we should create interdisciplinary environments on purpose to foster innovation, and this study’s results agree with that suggestion [41]. Furthermore, the percentage of scholars from an interdisciplinary field or a field outside of biology could be translated into a message that the funding agencies should prioritize the funding of interdisciplinary projects and institutions, because they may better lead the starting of a new discipline, or make a higher impact on existing fields.

Our results should not be interpreted to mean that systems biology is abandoning the systems-oriented components like modeling or databases in recent years. The opposite is true. As mentioned in the beginning of the article, system biology is characterized by big data and modeling, and the integration of mechanistic and mathematical explanations is and still will be underway [42]. What we mean is that modeling has changed from being a primary research subject into tools that help reveal biological mechanisms or solving a real-life problem. Systems-oriented components still play a very important role, but more biological components are integrated and transform systems biology to align better with other biological disciplines.

Some limitations of this research include: First, bibliographic analysis only allowed us to pick the most highly cited references, because it can be assumed that high citations can be interpreted as high impact and high quality. Therefore, these highly cited articles are more interesting and some argue that they can shed some light on the trend of this field, but we admit that there is a possibility that they do not fully represent the entire field. Second, we carefully categorized those articles through close reading, and the application of machine learning technique to analyze a much larger number of articles. While this enabled us to overcome the limitation that we could not read them all and it corroborated the results of close reading, machine learning techniques have some limitations in it, and it still cannot have the accuracy of human reading.

## Supporting information

S1 Table 330 most highly cited references and their categories. (DOCX)

S2 Table The word list used to identify the categories of institutions. (DOCX)

S3 Table Machine learning results of topics using MALLET. (DOCX)

S4 Table The percentage for articles that contained each of the 20 topics. (DOCX)

S1 Text A detailed description of nine categories of research in systems biology. (DOCX)
