# Supplementary material for: From systems to biology: A computational analysis of the research articles on systems biology from 1992 to 2013
Source: PLoS One. 2018 Jul 25;13(7):e0200929. doi: 10.1371/journal.pone.0200929 (PMC6059489; doi:10.1371/journal.pone.0200929)
Supplement: S1 Table — (DOCX) [file pone.0200929.s001.docx]

Supplementary information 1

This table show the first author, year, journal title, category of research of the most highly cited references for each time slice.

| Time slice | First Author | Year | Journal | Category | Systems-oirented/Biology- oriented |
| --- | --- | --- | --- | --- | --- |
| 1992-1993 | Kauffman S | 1993 | ORIGINS ORDER SELF O | A book |  |
|  | Fell DA | 1992 | BIOCHEM J | Metabolic Flux Analysis | Systems-oriented |
|  | Mendes P | 1993 | COMPUT APPL BIOSCI | Software development | Systems-oriented |
|  | Dekoning W | 1992 | ANAL BIOCHEM | Metabolic Flux Analysis | Systems-oriented |
|  | Gillespie DT | 1992 | PHYSICA A | Algorithms, equations, modeling and simulation | Systems-oriented |
|  | Novak B | 1993 | J CELL SCI | Algorithms, equations, modeling and simulation | Systems-oriented |
|  | Press WH | 1992 | NUMERICAL RECIPES C | A book |  |
|  | Verduyn C | 1992 | YEAST | Metabolic Flux Analysis | Systems-oriented |
|  | Koza J R | 1992 | GENETIC PROGRAMMING | A book |  |
|  | Van Kampen N G | 1992 | STOCHASTIC PROCESSES | A book |  |
|  | Lauffenburger D A | 1993 | RECEPTORS MODELS BIN | A book |  |
|  | Lee RC | 1993 | CELL | Biological Mechanisms | Biology-oriented |
|  | Cooper GF | 1992 | MACH LEARN | Algorithms, equations, modeling and simulation | Systems-oriented |
|  | Sali A | 1993 | J MOL BIOL | Algorithms, equations, modeling and simulation | Systems-oriented |
|  | Theobald U | 1993 | ANAL BIOCHEM | Hard to tell |  |
|  | Ermentrout GB | 1993 | J THEOR BIOL | Algorithms, equations, modeling and simulation | Systems-oriented |
|  | Holmes E | 1992 | MOL PHARMACOL | Biological Mechanisms | Biology-oriented |
|  | Segel I H | 1993 | ENZYME KINETICS BEHA | A book |  |
|  | Varma A | 1993 | APPL ENVIRON MICROB | Metabolic Flux Analysis | Systems-oriented |
|  | Celada F | 1992 | IMMUNOL TODAY | Algorithms, equations, modeling and simulation | Systems-oriented |
|  | Wallimann T | 1992 | BIOCHEM J | Biological Mechanisms | Biology-oriented |
|  | Ptashne M | 1992 | GENETIC SWITCH PHAGE | A book |  |
|  | Avery L | 1992 | TRENDS GENET | Biological Mechanisms | Biology-oriented |
|  | Varma A | 1993 | J THEOR BIOL | Metabolic Flux Analysis | Systems-oriented |
|  | Gillespie DT | 1992 | MARKOV PROCESSES INT | A book |  |
|  | Lawrence CE | 1993 | SCIENCE | Algorithms, equations, modeling and simulation | Systems-oriented |
|  | Boguski MS | 1993 | NAT GENET | Database building and curation | Systems-oriented |
|  | Niederberger P | 1992 | BIOCHEM J | Metabolic Flux Analysis | Systems-oriented |
|  | Graner F | 1992 | PHYS REV LETT | Algorithms, equations, modeling and simulation | Systems-oriented |
|  | Gelman Andrew | 1992 | STAT SCI | Algorithms, equations, modeling and simulation | Systems-oriented |
| 1994-1995 | Benjamini Y | 1995 | J ROY STAT SOC B MET | Algorithms, equations, modeling and simulation | Systems-oriented |
|  | Schena M | 1995 | SCIENCE | Development of high-throughput technologies | Biology-oriented |
|  | Eng JK | 1994 | J AM SOC MASS SPECTR | Algorithms, equations, modeling and simulation | Systems-oriented |
|  | Varma A | 1994 | BIO-TECHNOL | Metabolic Flux Analysis | Systems-oriented |
|  | Varma A | 1994 | APPL ENVIRON MICROB | Metabolic Flux Analysis | Systems-oriented |
|  | Fleischmann RD | 1995 | SCIENCE | Development of high-throughput technologies | Biology-oriented |
|  | Nicholson JK | 1995 | ANAL CHEM | Development of high-throughput technologies | Biology-oriented |
|  | Velculescu VE | 1995 | SCIENCE | Development of high-throughput technologies | Biology-oriented |
|  | Marshall CJ | 1995 | CELL | Biological Mechanisms | Biology-oriented |
|  | Thompson JD | 1994 | NUCLEIC ACIDS RES | Software development | Systems-oriented |
|  | Murzin AG | 1995 | J MOL BIOL | Database building and curation | Systems-oriented |
|  | Strogatz S H | 1994 | NONLINEAR DYNAMICS C | A book |  |
|  | Mcadams HH | 1995 | SCIENCE | Algorithms, equations, modeling and simulation | Systems-oriented |
|  | Thomas R | 1995 | B MATH BIOL | Network properties | Systems-oriented |
|  | Goldbeter A | 1995 | P ROY SOC B-BIOL SCI | Algorithms, equations, modeling and simulation | Systems-oriented |
|  | Bray D | 1995 | NATURE | Biological Mechanisms | Biology-oriented |
|  | Chalfie M | 1994 | SCIENCE | Development of high-throughput technologies | Biology-oriented |
|  | Ljung L | 1994 | AUTOMATICA | Algorithms, equations, modeling and simulation | Systems-oriented |
|  | Bailey T L | 1994 | Proc Int Conf Intell Syst Mol Biol | Algorithms, equations, modeling and simulation | Systems-oriented |
|  | Schuster S | 1994 | J BIOL SYST | Algorithms, equations, modeling and simulation | Systems-oriented |
|  | Szyperski T | 1995 | EUR J BIOCHEM | Development of high-throughput technologies | Biology-oriented |
|  | Boyd S | 1994 | LINEAR MATRIX INEQUA | A book |  |
|  | Arkin A | 1995 | J PHYS CHEM-US | Algorithms, equations, modeling and simulation | Systems-oriented |
|  | Kacser H | 1995 | BIOCHEM SOC T | Metabolic Flux Analysis | Systems-oriented |
|  | Yates JR | 1995 | ANAL CHEM | Hard to tell |  |
|  | Fraser CM | 1995 | SCIENCE | Omics research characterizing a real biological system | Biology-oriented |
|  | Feinberg M | 1995 | ARCH RATION MECH AN | Network properties | Systems-oriented |
|  | Fell DA | 1995 | BIOCHEM J | Metabolic Flux Analysis | Systems-oriented |
|  | Priami C | 1995 | COMPUT J | Algorithms, equations, modeling and simulation | Systems-oriented |
|  | Luo CH | 1994 | CIRC RES | Algorithms, equations, modeling and simulation | Systems-oriented |
| 1996-1997 | Heinrich R | 1996 | REGULATION CELLULAR | A book |  |
|  | Barkai N | 1997 | NATURE | Network properties | Systems-oriented |
|  | Altschul SF | 1997 | NUCLEIC ACIDS RES | Software development | Systems-oriented |
|  | Huang CYF | 1996 | P NATL ACAD SCI USA | Algorithms, equations, modeling and simulation | Systems-oriented |
|  | Mcadams HH | 1997 | P NATL ACAD SCI USA | Hard to tell |  |
|  | Derisi JL | 1997 | SCIENCE | Omics research characterizing a real biological system | Biology-oriented |
|  | Fell DA | 1997 | UNDERSTANDING CONTRO | A book |  |
|  | Blattner FR | 1997 | SCIENCE | Omics research characterizing a real biological system | Biology-oriented |
|  | Goffeau A | 1996 | SCIENCE | Omics research characterizing a real biological system | Biology-oriented |
|  | Lockhart DJ | 1996 | NAT BIOTECHNOL | Development of high-throughput technologies | Biology-oriented |
|  | Unlu M | 1997 | ELECTROPHORESIS | Development of high-throughput technologies | Systems-oriented |
|  | Tibshirani R | 1996 | J ROY STAT SOC B MET | Algorithms, equations, modeling and simulation | Systems-oriented |
|  | Mendes P | 1997 | TRENDS BIOCHEM SCI | Software development | Systems-oriented |
|  | Ferrell JE | 1996 | TRENDS BIOCHEM SCI | Biological Mechanisms | Systems-oriented |
|  | Rizzi M | 1997 | BIOTECHNOL BIOENG | Biological Mechanisms | Biology-oriented |
|  | Theobald U | 1997 | BIOTECHNOL BIOENG | Algorithms, equations, modeling and simulation | Systems-oriented |
|  | Arkin A | 1997 | SCIENCE | Algorithms, equations, modeling and simulation | Systems-oriented |
|  | Tatusov RL | 1997 | SCIENCE | Omics research characterizing a real biological system | Biology-oriented |
|  | Shevchenko A | 1996 | ANAL CHEM | Development of high-throughput technologies | Biology-oriented |
|  | Walter E | 1997 | IDENTIFICATION PARAM | A book |  |
|  | Goldbeter A | 1996 | BIOCH OSCILLATIONS C | A book |  |
|  | Bakker BM | 1997 | J BIOL CHEM | Metabolic Flux Analysis | Systems-oriented |
|  | Bonarius HPJ | 1997 | TRENDS BIOTECHNOL | Metabolic Flux Analysis | Systems-oriented |
|  | Wilkins MR | 1996 | BIOTECHNOL GENET ENG | Omics research characterizing a real biological system | Biology-oriented |
|  | Pramanik J | 1997 | BIOTECHNOL BIOENG | Metabolic Flux Analysis | Systems-oriented |
|  | Anderson L | 1997 | ELECTROPHORESIS | Omics research characterizing a real biological system | Biology-oriented |
|  | Reddy VN | 1996 | COMPUT BIOL MED | Metabolic Flux Analysis | Systems-oriented |
|  | Lutz R | 1997 | NUCLEIC ACIDS RES | Biological Mechanisms | Biology-oriented |
|  | Gonzalez B | 1997 | YEAST | Development of high-throughput technologies | Biology-oriented |
|  | Shevchenko A | 1996 | P NATL ACAD SCI USA | Development of high-throughput technologies | Biology-oriented |
| 1998-1999 | Nicholson JK | 1999 | XENOBIOTICA | Omics research characterizing a real biological system | Biology-oriented |
|  | Eisen MB | 1998 | P NATL ACAD SCI USA | Development of high-throughput technologies | Biology-oriented |
|  | Barabasi AL | 1999 | SCIENCE | Network properties | Systems-oriented |
|  | Hartwell LH | 1999 | NATURE | Biological Mechanisms | Biology-oriented |
|  | Gygi SP | 1999 | NAT BIOTECHNOL | Development of high-throughput technologies | Biology-oriented |
|  | Bhalla US | 1999 | SCIENCE | Network properties | Systems-oriented |
|  | Oliver SG | 1998 | TRENDS BIOTECHNOL | Omics research characterizing a real biological system | Biology-oriented |
|  | Arkin A | 1998 | GENETICS | Algorithms, equations, modeling and simulation | Systems-oriented |
|  | Watts DJ | 1998 | NATURE | Network properties | Systems-oriented |
|  | Spellman PT | 1998 | MOL BIOL CELL | Omics research characterizing a real biological system | Biology-oriented |
|  | Ogata H | 1999 | NUCLEIC ACIDS RES | Database building and curation | Systems-oriented |
|  | Mendes P | 1998 | BIOINFORMATICS | Algorithms, equations, modeling and simulation | Systems-oriented |
|  | Alon U | 1999 | NATURE | Network properties | Systems-oriented |
|  | Gygi SP | 1999 | MOL CELL BIOL | Omics research characterizing a real biological system | Biology-oriented |
|  | Tomita M | 1999 | BIOINFORMATICS | Software development | Systems-oriented |
|  | Kholodenko BN | 1999 | J BIOL CHEM | Algorithms, equations, modeling and simulation | Systems-oriented |
|  | Winzeler EA | 1999 | SCIENCE | Omics research characterizing a real biological system | Biology-oriented |
|  | Golub TR | 1999 | SCIENCE | The application of systems biology in the medical field. | Biology-oriented |
|  | Perkins DN | 1999 | ELECTROPHORESIS | Algorithms, equations, modeling and simulation | Systems-oriented |
|  | Stephanopoulos GN | 1998 | METABOLIC ENG PRINCI | A book |  |
|  | Rigaut G | 1999 | NAT BIOTECHNOL | Development of high-throughput technologies | Biology-oriented |
|  | Kohn KW | 1999 | MOL BIOL CELL | Omics research characterizing a real biological system | Biology-oriented |
|  | Ferrell JE | 1998 | SCIENCE | Biological Mechanisms | Biology-oriented |
|  | Mcadams HH | 1999 | TRENDS GENET | Network properties | Biology-oriented |
|  | Schuster S | 1999 | TRENDS BIOTECHNOL | Metabolic Flux Analysis | Systems-oriented |
|  | Link AJ | 1999 | NAT BIOTECHNOL | Development of high-throughput technologies | Biology-oriented |
|  | Pellegrini M | 1999 | P NATL ACAD SCI USA | Omics research characterizing a real biological system | Biology-oriented |
|  | Ljung L | 1999 | SYSTEM IDENTIFICATIO | A book |  |
|  | Tweeddale H | 1998 | J BACTERIOL | Omics research characterizing a real biological system | Biology-oriented |
|  | Marcotte EM | 1999 | SCIENCE | Omics research characterizing a real biological system | Biology-oriented |
| 2000-2001 | Ashburner M | 2000 | NAT GENET | Database building and curation | Systems-oriented |
|  | Ideker T | 2001 | ANNU REV GENOM HUM G | Hard to tell | Biology-oriented |
|  | Kanehisa M | 2000 | NUCLEIC ACIDS RES | Database building and curation | Systems-oriented |
|  | Uetz P | 2000 | NATURE | Omics research characterizing a real biological system | Biology-oriented |
|  | Ideker T | 2001 | SCIENCE | Omics research characterizing a real biological system | Biology-oriented |
|  | Jeong H | 2001 | NATURE | Network properties | Systems-oriented |
|  | Jeong H | 2000 | NATURE | Omics research characterizing a real biological system | Systems-oriented |
|  | Elowitz MB | 2000 | NATURE | Algorithms, equations, modeling and simulation | Systems-oriented |
|  | Fiehn O | 2000 | NAT BIOTECHNOL | Omics research characterizing a real biological system | Biology-oriented |
|  | Ito T | 2001 | P NATL ACAD SCI USA | Omics research characterizing a real biological system | Biology-oriented |
|  | Albert R | 2000 | NATURE | Network properties | Systems-oriented |
|  | Hanahan D | 2000 | CELL | The application of systems biology in the medical field. | Biology-oriented |
|  | Raamsdonk LM | 2001 | NAT BIOTECHNOL | Omics research characterizing a real biological system | Biology-oriented |
|  | Brazma A | 2001 | NAT GENET | Database building and curation | Systems-oriented |
|  | Gardner TS | 2000 | NATURE | The application of systems biology in the medical field. | Biology-oriented |
|  | Venter JC | 2001 | SCIENCE | Omics research characterizing a real biological system | Biology-oriented |
|  | Lander ES | 2001 | NATURE | Omics research characterizing a real biological system | Biology-oriented |
|  | Edwards JS | 2001 | NAT BIOTECHNOL | Omics research characterizing a real biological system | Systems-oriented |
|  | Friedman N | 2000 | J COMPUT BIOL | Algorithms, equations, modeling and simulation | Systems-oriented |
|  | Edwards JS | 2000 | P NATL ACAD SCI USA | Algorithms, equations, modeling and simulation | Systems-oriented |
|  | Teusink B | 2000 | EUR J BIOCHEM | Algorithms, equations, modeling and simulation | Systems-oriented |
|  | Tusher VG | 2001 | P NATL ACAD SCI USA | Omics research characterizing a real biological system | Biology-oriented |
|  | Von Dassow G | 2000 | NATURE | Network properties | Biology-oriented |
|  | Schuster S | 2000 | NAT BIOTECHNOL | Algorithms, equations, modeling and simulation | Systems-oriented |
|  | Gasch AP | 2000 | MOL BIOL CELL | Omics research characterizing a real biological system | Biology-oriented |
|  | Yi TM | 2000 | P NATL ACAD SCI USA | Algorithms, equations, modeling and simulation | Systems-oriented |
|  | Hughes TR | 2000 | CELL | Omics research characterizing a real biological system | Biology-oriented |
|  | Zhu H | 2001 | SCIENCE | Development of high-throughput technologies | Biology-oriented |
|  | Washburn MP | 2001 | NAT BIOTECHNOL | Omics research characterizing a real biological system | Biology-oriented |
|  | Voit E O | 2000 | COMPUTATIONAL ANAL B | A book |  |
| 2002-2003 | Kitano H | 2002 | SCIENCE | Hard to tell |  |
|  | Hucka M | 2003 | BIOINFORMATICS | Algorithms, equations, modeling and simulation | Systems-oriented |
|  | Shannon P | 2003 | GENOME RES | Software development | Systems-oriented |
|  | Kitano H | 2002 | NATURE | Omics research characterizing a real biological system | Systems-oriented |
|  | Fiehn O | 2002 | PLANT MOL BIOL | Omics research characterizing a real biological system | Biology-oriented |
|  | Milo R | 2002 | SCIENCE | Network properties | Systems-oriented |
|  | Lee TI | 2002 | SCIENCE | Omics research characterizing a real biological system | Biology-oriented |
|  | Ho Y | 2002 | NATURE | Omics research characterizing a real biological system | Biology-oriented |
|  | Nicholson JK | 2003 | NAT REV DRUG DISCOV | Omics research characterizing a real biological system | Biology-oriented |
|  | Aebersold R | 2003 | NATURE | Development of high-throughput technologies | Biology-oriented |
|  | Elowitz MB | 2002 | SCIENCE | Hard to tell |  |
|  | Shen-orr SS | 2002 | NAT GENET | Network properties | Systems-oriented |
|  | Gavin AC | 2002 | NATURE | Omics research characterizing a real biological system | Biology-oriented |
|  | Schoeberl B | 2002 | NAT BIOTECHNOL | Algorithms, equations, modeling and simulation | Systems-oriented |
|  | Ravasz E | 2002 | SCIENCE | Algorithms, equations, modeling and simulation | Systems-oriented |
|  | De Jong H | 2002 | J COMPUT BIOL | Algorithms, equations, modeling and simulation | Systems-oriented |
|  | Ghaemmaghami S | 2003 | NATURE | Omics research characterizing a real biological system | Biology-oriented |
|  | Giot L | 2003 | SCIENCE | Omics research characterizing a real biological system | Biology-oriented |
|  | Tyson JJ | 2003 | CURR OPIN CELL BIOL | Algorithms, equations, modeling and simulation | Systems-oriented |
|  | Peri S | 2003 | GENOME RES | Database building and curation | Systems-oriented |
|  | Csete ME | 2002 | SCIENCE | Algorithms, equations, modeling and simulation | Systems-oriented |
|  | Von Mering C | 2002 | NATURE | Omics research characterizing a real biological system | Biology-oriented |
|  | Forster J | 2003 | GENOME RES | Omics research characterizing a real biological system | Systems-oriented |
|  | Segre D | 2002 | P NATL ACAD SCI USA | Algorithms, equations, modeling and simulation | Systems-oriented |
|  | Nicholson JK | 2002 | NAT REV DRUG DISCOV | Omics research characterizing a real biological system | Biology-oriented |
|  | Ong SE | 2002 | MOL CELL PROTEOMICS | Development of high-throughput technologies | Biology-oriented |
|  | Giaever G | 2002 | NATURE | Omics research characterizing a real biological system | Biology-oriented |
|  | Gardner TS | 2003 | SCIENCE | Algorithms, equations, modeling and simulation | Systems-oriented |
|  | Segal E | 2003 | NAT GENET | Omics research characterizing a real biological system | Biology-oriented |
|  | Weckwerth W | 2003 | ANNU REV PLANT BIOL | Omics research characterizing a real biological system | Systems-oriented |
| 2004-2005 | Barabasi AL | 2004 | NAT REV GENET | Network properties | Systems-oriented |
|  | Hood L | 2004 | SCIENCE | The application of systems biology in the medical field. | Biology-oriented |
|  | Subramanian A | 2005 | P NATL ACAD SCI USA | Algorithms, equations, modeling and simulation | Systems-oriented |
|  | Rual JF | 2005 | NATURE | Omics research characterizing a real biological system | Biology-oriented |
|  | Stelzl U | 2005 | CELL | Omics research characterizing a real biological system | Biology-oriented |
|  | Han JDJ | 2004 | NATURE | Network properties | Systems-oriented |
|  | Price ND | 2004 | NAT REV MICROBIOL | Algorithms, equations, modeling and simulation | Systems-oriented |
|  | Kitano H | 2004 | NAT REV GENET | Network properties | Systems-oriented |
|  | Kell DB | 2004 | CURR OPIN MICROBIOL | Omics research characterizing a real biological system | Systems-oriented |
|  | Li SM | 2004 | SCIENCE | Omics research characterizing a real biological system | Biology-oriented |
|  | Goodacre R | 2004 | TRENDS BIOTECHNOL | Omics research characterizing a real biological system | Systems-oriented |
|  | Butcher EC | 2004 | NAT BIOTECHNOL | The application of systems biology in the medical field. | Biology-oriented |
|  | Westerhoff HV | 2004 | NAT BIOTECHNOL | The application of systems biology in the medical field. | Biology-oriented |
|  | Gentleman RC | 2004 | GENOME BIOL | Software development | Systems-oriented |
|  | Weston AD | 2004 | J PROTEOME RES | The application of systems biology in the medical field. | Biology-oriented |
|  | Sachs K | 2005 | SCIENCE | Algorithms, equations, modeling and simulation | Systems-oriented |
|  | Le Novere N | 2005 | NAT BIOTECHNOL | Algorithms, equations, modeling and simulation | Systems-oriented |
|  | Tong AHY | 2004 | SCIENCE | Omics research characterizing a real biological system | Biology-oriented |
|  | Stelling J | 2004 | CELL | Network properties | Systems-oriented |
|  | Covert MW | 2004 | NATURE | Algorithms, equations, modeling and simulation | Systems-oriented |
|  | Kitano H | 2005 | NAT BIOTECHNOL | Software development | Systems-oriented |
|  | Salwinski L | 2004 | NUCLEIC ACIDS RES | Database building and curation | Systems-oriented |
|  | Harbison CT | 2004 | NATURE | Omics research characterizing a real biological system | Biology-oriented |
|  | Fernie AR | 2004 | NAT REV MOL CELL BIO | Omics research characterizing a real biological system | Biology-oriented |
|  | Basso K | 2005 | NAT GENET | Algorithms, equations, modeling and simulation | Systems-oriented |
|  | Aderem A | 2005 | CELL | Hard to tell | Hard to tell |
|  | Ross PL | 2004 | MOL CELL PROTEOMICS | Development of high-throughput technologies | Biology-oriented |
|  | Joshi-tope G | 2005 | NUCLEIC ACIDS RES | Database building and curation | Systems-oriented |
|  | Maere S | 2005 | BIOINFORMATICS | Software development | Systems-oriented |
|  | Dunn WB | 2005 | ANALYST | Development of high-throughput technologies | Biology-oriented |
| 2006-2007 | Duarte NC | 2007 | P NATL ACAD SCI USA | Omics research characterizing a real biological system | Systems-oriented |
|  | Goh KI | 2007 | P NATL ACAD SCI USA | The application of systems biology in the medical field. | Biology-oriented |
|  | Hoops S | 2006 | BIOINFORMATICS | Software development | Systems-oriented |
|  | Krogan NJ | 2006 | NATURE | Omics research characterizing a real biological system | Biology-oriented |
|  | Gavin AC | 2006 | NATURE | Omics research characterizing a real biological system | Systems-oriented |
|  | Kanehisa M | 2006 | NUCLEIC ACIDS RES | Database building and curation | Systems-oriented |
|  | Alon U | 2007 | NAT REV GENET | The application of systems biology in the medical field. | Systems-oriented |
|  | Chuang HY | 2007 | MOL SYST BIOL | The application of systems biology in the medical field. | Biology-oriented |
|  | Gutenkunst RN | 2007 | PLOS COMPUT BIOL | Algorithms, equations, modeling and simulation | Systems-oriented |
|  | Stark C | 2006 | NUCLEIC ACIDS RES | Database building and curation | Systems-oriented |
|  | Le Novere N | 2006 | NUCLEIC ACIDS RES | Database building and curation | Systems-oriented |
|  | Wishart DS | 2007 | NUCLEIC ACIDS RES | Database building and curation | Systems-oriented |
|  | Bruggeman FJ | 2007 | TRENDS MICROBIOL | Hard to tell | Hard to tell |
|  | Cline MS | 2007 | NAT PROTOC | Software development | Systems-oriented |
|  | Kholodenko BN | 2006 | NAT REV MOL CELL BIO | Algorithms, equations, modeling and simulation | Systems-oriented |
|  | Joyce AR | 2006 | NAT REV MOL CELL BIO | Omics research characterizing a real biological system | Biology-oriented |
|  | Aldridge BB | 2006 | NAT CELL BIOL | Algorithms, equations, modeling and simulation | Systems-oriented |
|  | Feist AM | 2007 | MOL SYST BIOL | Omics research characterizing a real biological system | Biology-oriented |
|  | Lamb J | 2006 | SCIENCE | The application of systems biology in the medical field. | Biology-oriented |
|  | Margolin AA | 2006 | BMC BIOINFORMATICS | Algorithms, equations, modeling and simulation | Systems-oriented |
|  | Becker SA | 2007 | NAT PROTOC | Software development | Systems-oriented |
|  | Palsson B | 2006 | SYSTEMS BIOL PROPERT | A book |  |
|  | Clayton TA | 2006 | NATURE | The application of systems biology in the medical field. | Biology-oriented |
|  | Hornberg JJ | 2006 | BIOSYSTEMS | The application of systems biology in the medical field. | Biology-oriented |
|  | Kell DB | 2006 | DRUG DISCOV TODAY | The application of systems biology in the medical field. | Systems-oriented |
|  | Schmidt H | 2006 | BIOINFORMATICS | Software development | Systems-oriented |
|  | Bansal M | 2007 | MOL SYST BIOL | Algorithms, equations, modeling and simulation | Systems-oriented |
|  | Kerrien S | 2007 | NUCLEIC ACIDS RES | Database building and curation | Systems-oriented |
|  | Yildirim MA | 2007 | NAT BIOTECHNOL | The application of systems biology in the medical field. | Biology-oriented |
|  | Ishii N | 2007 | SCIENCE | Omics research characterizing a real biological system | Biology-oriented |
| 2008-2009 | Herrgard MJ | 2008 | NAT BIOTECHNOL | Algorithms, equations, modeling and simulation | Systems-oriented |
|  | Kanehisa M | 2008 | NUCLEIC ACIDS RES | Database building and curation | Systems-oriented |
|  | Nicholson JK | 2008 | NATURE | Omics research characterizing a real biological system | Biology-oriented |
|  | Feist AM | 2009 | NAT REV MICROBIOL | Algorithms, equations, modeling and simulation | Biology-oriented |
|  | Le Novere N | 2009 | NAT BIOTECHNOL | Algorithms, equations, modeling and simulation | Systems-oriented |
|  | Feist AM | 2008 | NAT BIOTECHNOL | Omics research characterizing a real biological system | Systems-oriented |
|  | Wang Z | 2009 | NAT REV GENET | Development of high-throughput technologies | Biology-oriented |
|  | Yu HY | 2008 | SCIENCE | Omics research characterizing a real biological system | Biology-oriented |
|  | Hopkins AL | 2008 | NAT CHEM BIOL | The application of systems biology in the medical field. | Biology-oriented |
|  | Oberhardt MA | 2009 | MOL SYST BIOL | Hard to tell |  |
|  | Querec TD | 2009 | NAT IMMUNOL | The application of systems biology in the medical field. | Biology-oriented |
|  | Jensen LJ | 2009 | NUCLEIC ACIDS RES | Database building and curation | Systems-oriented |
|  | Chen YQ | 2008 | NATURE | The application of systems biology in the medical field. | Biology-oriented |
|  | Sreekumar A | 2009 | NATURE | The application of systems biology in the medical field. | Biology-oriented |
|  | Shlomi T | 2008 | NAT BIOTECHNOL | Algorithms, equations, modeling and simulation | Systems-oriented |
|  | Mortazavi A | 2008 | NAT METHODS | Hard to tell |  |
|  | Prasad TSK | 2009 | NUCLEIC ACIDS RES | Database building and curation | Systems-oriented |
|  | Wishart DS | 2009 | NUCLEIC ACIDS RES | Database building and curation | Systems-oriented |
|  | Holmes E | 2008 | NATURE | The application of systems biology in the medical field. | Biology-oriented |
|  | Matthews L | 2009 | NUCLEIC ACIDS RES | Database building and curation | Systems-oriented |
|  | Picotti P | 2009 | CELL | Omics research characterizing a real biological system | Biology-oriented |
|  | Chen WW | 2009 | MOL SYST BIOL | Algorithms, equations, modeling and simulation | Systems-oriented |
|  | Campillos M | 2008 | SCIENCE | The application of systems biology in the medical field. | Biology-oriented |
|  | Breitkreutz BJ | 2008 | NUCLEIC ACIDS RES | Database building and curation | Systems-oriented |
|  | Hillenmeyer ME | 2008 | SCIENCE | Omics research characterizing a real biological system | Biology-oriented |
|  | Emilsson V | 2008 | NATURE | The application of systems biology in the medical field. | Biology-oriented |
|  | Pico AR | 2008 | PLOS BIOL | Database building and curation | Systems-oriented |
|  | Caspi R | 2008 | NUCLEIC ACIDS RES | Database building and curation | Systems-oriented |
|  | Ideker T | 2008 | GENOME RES | The application of systems biology in the medical field. | Biology-oriented |
|  | Zhu J | 2008 | NAT GENET | Omics research characterizing a real biological system | Systems-oriented |
| 2010-2011 | Kanehisa M | 2010 | NUCLEIC ACIDS RES | Database building and curation | Systems-oriented |
|  | Barabasi AL | 2011 | NAT REV GENET | The application of systems biology in the medical field. | Biology-oriented |
|  | Thiele I | 2010 | NAT PROTOC | Hard to tell |  |
|  | Smoot ME | 2011 | BIOINFORMATICS | Software development | Systems-oriented |
|  | Orth JD | 2010 | NAT BIOTECHNOL | Metabolic Flux Analysis | Systems-oriented |
|  | Szklarczyk D | 2011 | NUCLEIC ACIDS RES | Database building and curation | Systems-oriented |
|  | Hanahan D | 2011 | CELL | The application of systems biology in the medical field. | Biology-oriented |
|  | Costanzo M | 2010 | SCIENCE | Omics research characterizing a real biological system | Biology-oriented |
|  | Gehlenborg N | 2010 | NAT METHODS | Omics research characterizing a real biological system | Systems-oriented |
|  | Vidal M | 2011 | CELL | The application of systems biology in the medical field. | Biology-oriented |
|  | Demir E | 2010 | NAT BIOTECHNOL | Database building and curation | Systems-oriented |
|  | Nakaya HI | 2011 | NAT IMMUNOL | The application of systems biology in the medical field. | Biology-oriented |
|  | Li C | 2010 | BMC SYST BIOL | Database building and curation | Systems-oriented |
|  | Schwanhausser B | 2011 | NATURE | Omics research characterizing a real biological system | Biology-oriented |
|  | Kreeger PK | 2010 | CARCINOGENESIS | The application of systems biology in the medical field. | Biology-oriented |
|  | Henry CS | 2010 | NAT BIOTECHNOL | Algorithms, equations, modeling and simulation | Systems-oriented |
|  | Saito K | 2010 | ANNU REV PLANT BIOL | Omics research characterizing a real biological system | Biology-oriented |
|  | Aranda B | 2010 | NUCLEIC ACIDS RES | Database building and curation | Systems-oriented |
|  | Kohl P | 2010 | CLIN PHARMACOL THER | Hard to tell |  |
|  | Stark C | 2011 | NUCLEIC ACIDS RES | Database building and curation | Systems-oriented |
|  | Croft D | 2011 | NUCLEIC ACIDS RES | Database building and curation | Systems-oriented |
|  | Qin JJ | 2010 | NATURE | Omics research characterizing a real biological system | Biology-oriented |
|  | Marbach D | 2010 | P NATL ACAD SCI USA | Algorithms, equations, modeling and simulation | Systems-oriented |
|  | Schellenberger J | 2010 | BMC BIOINFORMATICS | Database building and curation | Systems-oriented |
|  | Breitkreutz A | 2010 | SCIENCE | Omics research characterizing a real biological system | Biology-oriented |
|  | Metzker ML | 2010 | NAT REV GENET | Development of high-throughput technologies | Biology-oriented |
|  | Caspi R | 2010 | NUCLEIC ACIDS RES | Database building and curation | Systems-oriented |
|  | Chuang HY | 2010 | ANNU REV CELL DEV BI | Hard to tell |  |
|  | Pulendran B | 2010 | IMMUNITY | The application of systems biology in the medical field. | Biology-oriented |
|  | Jerby L | 2010 | MOL SYST BIOL | Hard to tell |  |
| 2012-2013 | Kanehisa M | 2012 | NUCLEIC ACIDS RES | Database building and curation | Systems-oriented |
|  | Chen R | 2012 | CELL | The application of systems biology in the medical field. | Biology-oriented |
|  | Ideker T | 2012 | MOL SYST BIOL | Algorithms, equations, modeling and simulation | Systems-oriented |
|  | Caspi R | 2012 | NUCLEIC ACIDS RES | Database building and curation | Systems-oriented |
|  | Karr JR | 2012 | CELL | Algorithms, equations, modeling and simulation | Systems-oriented |
|  | Kerrien S | 2012 | NUCLEIC ACIDS RES | Database building and curation | Systems-oriented |
|  | Lewis NE | 2012 | NAT REV MICROBIOL | Algorithms, equations, modeling and simulation | Systems-oriented |
|  | Nicholson JK | 2012 | SCIENCE | The application of systems biology in the medical field. | Biology-oriented |
|  | Patti GJ | 2012 | NAT REV MOL CELL BIO | Omics research characterizing a real biological system | Biology-oriented |
|  | Lee JW | 2012 | NAT CHEM BIOL | The application of systems biology in the medical field. | Biology-oriented |
|  | Dunham I | 2012 | NATURE | Omics research characterizing a real biological system | Biology-oriented |
|  | Marbach D | 2012 | NAT METHODS | Algorithms, equations, modeling and simulation | Systems-oriented |
|  | Zhang AH | 2012 | ANALYST | Development of high-throughput technologies | Biology-oriented |
|  | Zhao S | 2012 | ANNU REV PHARMACOL | The application of systems biology in the medical field. | Biology-oriented |
|  | Licata L | 2012 | NUCLEIC ACIDS RES | Database building and curation | Systems-oriented |
|  | Punta M | 2012 | NUCLEIC ACIDS RES | Database building and curation | Systems-oriented |
|  | Barretina J | 2012 | NATURE | The application of systems biology in the medical field. | Biology-oriented |
|  | Wang XJ | 2012 | NAT BIOTECHNOL | The application of systems biology in the medical field. | Biology-oriented |
|  | Lee MJ | 2012 | CELL | The application of systems biology in the medical field. | Biology-oriented |
|  | Picotti P | 2012 | NAT METHODS | Omics research characterizing a real biological system | Biology-oriented |
|  | Cherry JM | 2012 | NUCLEIC ACIDS RES | Database building and curation | Database building and curation |
|  | Huttenhower C | 2012 | NATURE | The application of systems biology in the medical field. | Biology-oriented |
|  | Zhang AH | 2012 | J PROTEOMICS | Omics research characterizing a real biological system | Biology-oriented |
|  | Wang XJ | 2012 | MOL CELL PROTEOMICS | The application of systems biology in the medical field. | Biology-oriented |
|  | Gerlinger M | 2012 | NEW ENGL J MED | The application of systems biology in the medical field. | Biology-oriented |
|  | Zhang AH | 2012 | COMPLEMENT THER MED | The application of systems biology in the medical field. | Biology-oriented |
|  | Hood L | 2012 | NEW BIOTECHNOL | The application of systems biology in the medical field. | Biology-oriented |
|  | He JC | 2012 | KIDNEY INT | The application of systems biology in the medical field. | Biology-oriented |
|  | Vanlier J | 2012 | BIOINFORMATICS | Algorithms, equations, modeling and simulation | Systems-oriented |
|  | Kelder T | 2012 | NUCLEIC ACIDS RES | Database building and curation | Systems-oriented |
