# Supplementary material for: From systems to biology: A computational analysis of the research articles on systems biology from 1992 to 2013
Source: PLoS One. 2018 Jul 25;13(7):e0200929. doi: 10.1371/journal.pone.0200929 (PMC6059489; doi:10.1371/journal.pone.0200929)
Supplement: S2 Table — (DOCX) [file pone.0200929.s002.docx]

Supporting information 3

The table shows four categories of words that are used to automatically match the addresses of authors to identify four types of institutions: biology-oriented, systems-oriented, interdisciplinary, and systems biology institutes.

| Four Categories | Words |
| --- | --- |
| Words for biology-oriented institutions (119 words) | allergy, anim, animal, anat, anatomy, anesthesiology, anesthesiol, arteriosclerosis, bioanalyt, biometry, biosci, brain, bacteriol, conservat, cattle, cardiol, canc, cancer, cardiac, cardiovasc, cell, clin, clinical, cytology, cytol, dermatol, dermatology, developmental, diseases, dna, drug, ecol, ecology, entomol, entomology, epidemiology, evolutionary, food, genet, genetics, genom, genome, genomics, glaxosmithkline, health, heart, hlth, hosp, hospital, Hepatol, Hepatology, human, immunology, immunol, Immunotechnol, immun, infect, insect, life, liver, livestock, lung, marine, med, medical, medicine, merck, microbiol, microbial, microbiology, mol, molecular, neurosci, nih, neurology, neurol, nutrit, nutrition, nutr, oncology, oncol, oral, organ, pfizer, pediat, pediatric, pathol, pathology, padiat, pediatrics, plant, pharm, pharma, pharmacol, pharmacology, pharmaceut, physiol, physiology, plant, proteomics, psychiat, reprod, reproduce, structrual, surgery, surg, syngenta, therapeut, therapeutical, toxicol, toxicology, vaccine, vaccines, vet, virol, virus, virology, zoology, zool |
| Words for systems-oriented institutions (47 words) | artificial, artificial, astrophysics, aerosp, aerospace, automat, chem, chem, chemical, chemical, chemistry, commun, communication, comp, computat, computer, cs, control, data, database , dynam, dynamics, elect, elec, ee, energy, electrical, infocomm, informat, information, mat, math, mathematics, mech, mechanical, microsoft, modeling, phys, physics, sensors, siemens, signals, simulat, sony, statistics, stat, weapons |
| Words for Interdisciplinary institutions (24 words) | biochem, biochemistry, biocomputing, biodynamics, biodesign, bioenergy, bioenerget, bioenergetics, bioengn, bioengineering, bioinformat, bioinformatics, biomech, biomechanics, biomodeling, biophysics, biophys, biostatistics, biostat, biotech, biotechnol, biotechnology, ebi, interdisciplinary |
| Systems biology institutions (3 words) | systems biology, biosystems, biosyst |
