# Supplementary material for: From systems to biology: A computational analysis of the research articles on systems biology from 1992 to 2013
Source: PLoS One. 2018 Jul 25;13(7):e0200929. doi: 10.1371/journal.pone.0200929 (PMC6059489; doi:10.1371/journal.pone.0200929)
Supplement: S3 Table — (DOCX) [file pone.0200929.s003.docx]

Supporting information 4

It shows the words in the 20 topic bins that are based on the machine learning of the topics in abstracts of 8809 articles on systems biology.

Most likely words found in the 20 topics of systems biology.

| Index | Most likely words in topic  (Machine Assigned) | Description of the topic  (Manually assigned) |
| --- | --- | --- |
| 0 | plant species molecular biology systems development processes plants physiological environment developmental life physiology major importance arabidopsis organisms environmental increasing | Biology |
| 1 | model models modeling computational experimental mathematical process simulation systems modelling system quantitative biological biochemical framework processes complex dynamic hypotheses | models |
| 2 | metabolites mass metabolomics ms samples spectrometry metabolite profiling metabolic high sample quantitative nmr analytical identification profiles metabolomic detection quantification | metabolic studies |
| 3 | metabolic metabolism growth conditions flux enzymes acid enzyme energy glucose yeast production rate mitochondrial strains coli pathway carbon strain | metabolic studies |
| 4 | human medicine health effects environmental individual impact toxicity risk personalized potential major current assessment exposure development disease chemical animal | disease |
| 5 | protein proteins interactions interaction molecular functional function structural complex proteome specific human functions complexes cellular proteomic molecules proteomics large | proteomics |
| 6 | systems biology design engineering metabolic scale genome production synthetic microbial process natural strategies products applications potential efficient development interest | synthetic biology |
| 7 | data information pathway tools integration database biological databases pathways software developed literature tool web large integrated resources facilitate open | database/software |
| 8 | cell cells single cellular high vivo quantitative individual molecules imaging time tissue spatial low intracellular small patterns tissues surface | Cell/tissue |
| 9 | systems biology biological complex molecular level system processes complexity view components context fundamental principles living concepts general theory perspective | systems theory |
| 10 | data parameters number experiments experimental set parameter time large algorithm sets statistical sensitivity applied algorithms prediction values inference predict | algorithms |
| 11 | response responses host immune mechanisms stress specific systems infection pathways biology cellular cells bacterial virus vaccine pathogen background understood | Immune systems |
| 12 | dynamics system control state time model reaction behavior stochastic differential reserved biochemical dynamic rate conditions cycle feedback kinetic reactions | dynamics and stochasticity |
| 13 | network networks regulatory biological interactions structure cellular complex scale components modules functions properties features robustness relationships multiple functional information | network |
| 14 | disease diseases tissue liver blood patients brain mice mechanisms disorders aging role heart human chronic normal induced increased tissues | disease |
| 15 | gene genes expression genetic genome functional identified microarray analyses data identify phenotypes genomic phenotype pathways expressed wide common specific | genomics |
| 16 | recent high technologies throughput field advances techniques biology proteomics omics genomics tools development years current future technology challenges applications | technologies and tools |
| 17 | drug cancer clinical targets discovery disease target treatment potential drugs molecular therapeutic development biomarkers tumor therapy diseases patients multiple | drug |
| 18 | regulation dna transcription regulatory gene transcriptional binding rna factors sequence expression mrna evolution genome msb specific sequences factor sites | regulation |
| 19 | cell signaling cells pathways pathway signal activation receptor signalling kinase growth beta transduction stem factor phosphorylation activity differentiation alpha | pathway |
