# Supplementary material for: From systems to biology: A computational analysis of the research articles on systems biology from 1992 to 2013
Source: PLoS One. 2018 Jul 25;13(7):e0200929. doi: 10.1371/journal.pone.0200929 (PMC6059489; doi:10.1371/journal.pone.0200929)
Supplement: S4 Table — (DOCX) [file pone.0200929.s004.docx]

Supporting information 5

It shows for each topic, the percentage of articles containing that topic over time.

The percentage of articles containing each topic over time.

|  | 2003 | 2004 | 2005 | 2006 | 2007 | 2008 | 2009 | 2010 | 2011 | 2012 | 2013 |
| --- | --- | --- | --- | --- | --- | --- | --- | --- | --- | --- | --- |
| Topic 0 | 0.118 | 0.082 | 0.092 | 0.045 | 0.063 | 0.049 | 0.069 | 0.069 | 0.078 | 0.068 | 0.072 |
| Topic 1 | 0.140 | 0.137 | 0.125 | 0.141 | 0.104 | 0.125 | 0.119 | 0.107 | 0.102 | 0.103 | 0.096 |
| Topic 2 | 0.075 | 0.073 | 0.111 | 0.107 | 0.129 | 0.114 | 0.092 | 0.072 | 0.094 | 0.090 | 0.080 |
| Topic 3 | 0.097 | 0.087 | 0.073 | 0.073 | 0.092 | 0.094 | 0.099 | 0.110 | 0.115 | 0.096 | 0.112 |
| Topic 4 | 0.151 | 0.078 | 0.095 | 0.068 | 0.054 | 0.080 | 0.066 | 0.080 | 0.073 | 0.096 | 0.078 |
| Topic 5 | 0.043 | 0.082 | 0.098 | 0.086 | 0.101 | 0.080 | 0.101 | 0.089 | 0.088 | 0.078 | 0.070 |
| Topic 6 | 0.075 | 0.078 | 0.060 | 0.064 | 0.053 | 0.058 | 0.067 | 0.068 | 0.069 | 0.072 | 0.074 |
| Topic 7 | 0.086 | 0.142 | 0.114 | 0.133 | 0.132 | 0.103 | 0.102 | 0.087 | 0.090 | 0.087 | 0.088 |
| Topic 8 | 0.043 | 0.050 | 0.060 | 0.103 | 0.061 | 0.071 | 0.066 | 0.067 | 0.075 | 0.072 | 0.076 |
| Topic 9 | 0.140 | 0.160 | 0.133 | 0.109 | 0.104 | 0.123 | 0.109 | 0.090 | 0.108 | 0.077 | 0.069 |
| Topic 10 | 0.086 | 0.073 | 0.079 | 0.111 | 0.101 | 0.100 | 0.107 | 0.100 | 0.088 | 0.107 | 0.104 |
| Topic 11 | 0.032 | 0.027 | 0.035 | 0.041 | 0.045 | 0.046 | 0.056 | 0.071 | 0.092 | 0.093 | 0.108 |
| Topic 12 | 0.118 | 0.132 | 0.119 | 0.116 | 0.107 | 0.099 | 0.104 | 0.106 | 0.103 | 0.091 | 0.088 |
| Topic 13 | 0.129 | 0.100 | 0.100 | 0.105 | 0.106 | 0.100 | 0.096 | 0.095 | 0.084 | 0.090 | 0.082 |
| Topic 14 | 0.086 | 0.059 | 0.046 | 0.068 | 0.094 | 0.113 | 0.093 | 0.121 | 0.121 | 0.132 | 0.145 |
| Topic 15 | 0.075 | 0.096 | 0.111 | 0.083 | 0.099 | 0.095 | 0.105 | 0.114 | 0.093 | 0.106 | 0.101 |
| Topic 16 | 0.161 | 0.169 | 0.173 | 0.182 | 0.145 | 0.155 | 0.142 | 0.111 | 0.116 | 0.118 | 0.116 |
| Topic 17 | 0.011 | 0.068 | 0.098 | 0.101 | 0.097 | 0.094 | 0.091 | 0.110 | 0.120 | 0.145 | 0.141 |
| Topic 18 | 0.054 | 0.050 | 0.060 | 0.083 | 0.092 | 0.063 | 0.093 | 0.093 | 0.083 | 0.087 | 0.072 |
| Topic 19 | 0.065 | 0.073 | 0.087 | 0.077 | 0.099 | 0.101 | 0.113 | 0.104 | 0.114 | 0.110 | 0.111 |
