# Supplementary material for: From systems to biology: A computational analysis of the research articles on systems biology from 1992 to 2013
Source: PLoS One. 2018 Jul 25;13(7):e0200929. doi: 10.1371/journal.pone.0200929 (PMC6059489; doi:10.1371/journal.pone.0200929)
Supplement: S1 Text — (DOCX) [file pone.0200929.s005.docx]

Supporting information 2

**Nine categories of research in systems biology**

1): Metabolic Flux Analysis

Metabolic Flux Analysis (MFA) measures the stoichiometric data of metabolites, and it relies on modeling using non-differential equations and a few parameters (Fell, 1992). Therefore, we classify these articles as systems-oriented. If one searches the term “systems biology” in these articles, one will not find any matching this criterion, but some have remarked that MFA is the early precursors of systems biology.

In early 1990s, many articles fall into this category. However, MFA becomes part of metabolomics which is classified into the category of “omics” research, and the name MFA is less frequently mentioned, so a decline of the slope for metabolic flux analysis is observed.

2): Development of high-throughput technologies

The high-throughput technologies include but are not limited to sequencing technologies, protein chips, DNA arrays, and biological measurements using Mass Spectrometry (Hood 2003). The focus of these articles in this category is on technologies *per se*. The preparation of technologies contributed greatly to the emergence of systems biology.

Most highly cited articles about the development of high-throughput technologies were published in early 1990s. Such studies decline in the latter half of the slope, mainly because the main technologies had been developed before 2005.

3): Algorithms, equations, modeling and simulation.

Without mathematical modeling, the data produced by high-throughput technologies would be meaningless. Mostly, mathematicians and engineers develop algorithms, equations, and modeling and simulation to infer or reconstruct metabolic pathways, signal transduction pathways, or gene regulatory networks (Brigandt, 2013). Articles which fall into this category are those which focus solely on algorithms and modeling *per se*.

4): Omics research characterizing a real biological system.

Omics research relies on the data produced by high-throughput technologies and modeling, but the ultimate goal is offering a system-level characterization of an organism (Joyce & Palsson, 2006). Metabolomics is part of omics that has Metabolic Flux Analysis its precursor. However, metabolomics measures the metabolites using Mass Spectrometry and other more advanced technologies; therefore, its goal is to systematically study all the metabolites and how they interact. This type of research began to emerge with the sequencing of genomes of several important model organisms. For example, the flu genome and the yeast genome were sequenced in 1995 and1996 respectively. Omics research gradually takes up a large percentage in early 2000s.

5): Database building and curation.

This category of research involves the launch of databases storing genes, pathways, proteins, etc. It also involves the standardization of data and curation procedures, such as the SBML (Systems Biology Markup Language) and KEGG. Since then, more and more other databases were developed, such as MINT (Molecular Interaction Database).

6): Software development.

Software is developed to process, analyze, and visualize large data and this category is straightforward. For example, Cytoscape is especially useful for mapping various biochemical networks and was released in 2002 (Shannon et al., 2002). The developers of Cytoscape include a group of computer scientists at the Institute of Systems Biology in Seattle and engineers in the Department of Bioengineering at UCSD, and biologists from Whitehead Institute for Biomedical Research. Cytoscape can be used to visualize according to different algorithms, and analyze the network, for example, giving measurements about the centrality of the nodes. Other software includes the OpenCOBRA project and COPASI (Hoops et al., 2006).

7): Theoretical and mathematical work on network properties

These properties include robustness, dynamics, stochasticity, and emergent properties of networks. These properties can be applied to every system, not just biological systems. These studies usually deploy mathematical models to study the network properties. Therefore, they are mostly systems-oriented. The study of these network properties is not a recent thing. For example, in 1997, Barkai and Leiber had already studied the robustness of simple biochemical networks (Barkal & Leiber, 1997).

8): The application of systems biology in the medical field.

Systems biology is especially useful in tackling complex diseases such as cancer, because scientists have realized that cancer has multiple causes. In the last ten years, cancer systems biology has made much progress such as building cancer genome databases and uncovering the regulatory networks underlining cancer. Another example is that in 2004, Leroy Hood proposed that systems biology will lead to predictive, preventive and personalized medicine, which is different from traditional medicine in that it will be produced with a systems understanding of the causes of disease (Weston & Hood, 2004). The application of systems biology is the most prominent feature of the advancements of the field in the last several years.

9): Biological mechanisms

This category of research involves using systems approach to understand a specific biological mechanism, for example, how FAR1 functions in the signal transduction pathway to link to the cell cycle machinery in yeast. The main focus is on revealing a biological mechanism (Peter, Gartner, Horecka, Ammerer, & Herskowitz, 1993).

References:

Fell, David A. (1992). Metabolic control analysis: a survey of its theoretical and experimental development. *Biochemical Journal*286, 2: 313-330.

Hood, L. (2003). Systems biology: integrating technology, biology, and computation. *Mechanisms of Ageing and Development*, 124(1), 9-16.

Brigandt, I. (2013). Systems biology and the integration of mechanistic explanation and mathematical explanation. *Studies in History and Philosophy of Science Part C: Studies in History and Philosophy of Biological and Biomedical Sciences,* 44(4), 477-492.

Joyce, A. R., & Palsson, B. Ø. (2006). The model organism as a system: integrating 'omics' data sets. *Nature Reviews Molecular Cell Biology*, *7*(3), 198-210.

Shannon, P., Markiel, A., Ozier, O., Baliga, N. S., Wang, J. T., Ramage, D., ... & Ideker, T. (2003). Cytoscape: a software environment for integrated models of biomolecular interaction networks. *Genome Research,* 13(11), 2498-2504.

Hoops, S., Sahle, S., Gauges, R., Lee, C., Pahle, J., Simus, N., ... & Kummer, U. (2006). COPASI—a complex pathway simulator. *Bioinformatics*, *22*(24), 3067-3074.

Barkal, N., & Leibler, S. (1997). Robustness in simple biochemical networks. *Nature*, 387(6636), 913-917.

Weston, A., & Hood, L. (2004). Systems biology, proteomics, and the future of health care: toward predictive, preventative, and personalized medicine*.* *Journal of Proteome Research*, 3(2), 179–196.

Peter, M., Gartner, A., Horecka, J., Ammerer, G., & Herskowitz, I. (1993). FAR1 links the signal transduction pathway to the cell cycle machinery in yeast. *Cell*, *73*(4), 747-760.
